# Pharmacological enhancement of autophagy mitigates tau pathology in a stem cell model

**DOI:** 10.1002/alz70861_108382

**Published:** 2025-12-23

**Authors:** Farzane S. Mirfakhar, Jacob Marsh, Miguel Minaya, Stephen Pak, Gary Silverman, David Perlmutter, Shannon L Macauley, Celeste M. Karch

**Affiliations:** ^1^ Washington University School of Medicine, St Louis, MO USA; ^2^ Washington University in St Louis, St Louis, MO USA; ^3^ Washington University in St Louis, Saint Louis, MO USA; ^4^ University of Kentucky College of Medicine, Sanders‐Brown Center on Aging, Lexington, KY USA; ^5^ University of Kentucky, Lexington, KY USA; ^6^ The Charles F. and Joanne Knight Alzheimer Disease Research Center, Washington University, St. Louis, MO USA; ^7^ Washington University in St. Louis, St. Louis, MO USA

## Abstract

**Background:**

Tau degradation is disrupted in neurodegenerative tauopathies, such as frontotemporal dementia (FTD), which may contribute to Tau aggregation. The prevailing hypothesis has been that Tau degradation is stymied due to an imbalance in proteostasis that occurs with age.

**Method:**

Here, we used Airyscan super resolution imaging to illustrate that a pathogenic FTD mutation in the *MAPT* gene, which encodes Tau, is sufficient to alter multiple steps of the autophagy lysosomal pathway and impair Tau degradation in stem cell derived neurons.

**Result:**

We discovered lysosomes clogged with both Tau and phosphorylated Tau, stalled lysosome motility, disrupted molecular motors, enhanced autophagic flux, and slowed cargo degradation in mutant Tau neurons. Treatment of mutant Tau neurons with a small molecule autophagy enhancer drug increases autophagic flux and cargo degradation, reduces phospho‐Tau levels, and reduces Tau accumulation in lysosomes without restoring defects in lysosomal motility.

**Conclusion:**

This study reveals novel effects of mutant Tau and provides a window through which therapeutic treatments targeting autophagy may promote Tau homeostasis.

